# Evaluation of Neutralizing Activity against Omicron Subvariants in BA.5 Breakthrough Infection and Three-Dose Vaccination Using a Novel Chemiluminescence-Based, Virus-Mediated Cytopathic Assay

**DOI:** 10.1128/spectrum.00660-23

**Published:** 2023-06-13

**Authors:** Mako Toyoda, Toong Seng Tan, Chihiro Motozono, Godfrey Barabona, Akiko Yonekawa, Nobuyuki Shimono, Rumi Minami, Yoji Nagasaki, Yusuke Miyashita, Hiroyuki Oshiumi, Kimitoshi Nakamura, Shuzo Matsushita, Takeo Kuwata, Takamasa Ueno

**Affiliations:** a Division of Infection and Immunity, Joint Research Center for Human Retrovirus Infection, Kumamoto University, Kumamoto, Japan; b Graduate School of Medical Sciences, Kumamoto University, Kumamoto, Japan; c Department of Medicine and Biosystemic Science, Graduate School of Medical Sciences, Kyushu University, Fukuoka, Japan; d Internal Medicine, Clinical Research Institute, National Hospital Organization, Kyushu Medical Center, Fukuoka, Japan; e Division of Infectious Diseases, Clinical Research Institute, National Hospitalization Organization, Kyushu Medical Center, Fukuoka, Japan; f Department of Immunology, Graduate School of Medical Sciences, Faculty of Life Sciences, Kumamoto University, Kumamoto, Japan; g Department of Pediatrics, Graduate School of Medical Sciences, Kumamoto University, Kumamoto, Japan; h Division of Clinical Retrovirology, Joint Research Center for Human Retrovirus Infection, Kumamoto University, Kumamoto, Japan; Technion—Israel Institute of Technology

**Keywords:** Omicron subvariants, SARS-CoV-2, cytopathic effect, neutralizing assay

## Abstract

Neutralizing potency of humoral immune responses induced by prior infection or vaccination is vital for protecting of individuals and population against severe acute respiratory syndrome-related coronavirus 2 (SARS-CoV-2). However, the emergence of viral variants that can evade neutralization by vaccine- or infection-induced immunity is a significant public health threat and requires continuous monitoring. Here, we have developed a novel scalable chemiluminescence-based assay for assessing SARS-CoV-2-induced cytopathic effect to quantify the neutralizing activity of antisera. The assay leverages the correlation between host cell viability and ATP levels in culture to measure the cytopathic effect on target cells induced by clinically isolated, replication-competent, authentic SARS-CoV-2. With this assay, we demonstrate that the recently arisen Omicron subvariants BQ.1.1 and XBB.1 display a significant decrease in sensitivity to neutralization by antibodies elicited from breakthrough infections with Omicron BA.5 and from receipt of three doses of mRNA vaccines. Thus, this scalable neutralizing assay provides a useful platform to assess the potency of acquired humoral immunity against newly emerging SARS-CoV-2 variants.

**IMPORTANCE** The ongoing global pandemic of SARS-CoV-2 has emphasized the importance of neutralizing immunity in protecting individuals and populations against severe respiratory illness. In light of the emergence of viral variants with the potential to evade immunity, continuous monitoring is imperative. A virus plaque reduction neutralization test (PRNT) is a “gold standard” assay for analyzing neutralizing activity for authentic viruses that form plaques, like influenza virus, dengue virus, and SARS-CoV-2. However, this method is labor intensive and is not efficient for performing large-scale neutralization assays on patient specimens. The assay system established in this study allows for the detection of a patient's neutralizing activity by simply adding an ATP detection reagent, providing a simple evaluation system for neutralizing activity of antisera as an alternative to the plaque reduction method. Our extended analysis of the Omicron subvariants highlights their increasing capability to evade neutralization by both vaccine- and infection-induced humoral immunity.

## OBSERVATION

The coronavirus disease 2019 (COVID-19) pandemic, caused by severe acute respiratory syndrome coronavirus 2 (SARS-CoV-2) has now caused over 6 million deaths worldwide (1). Despite the increased population immunity from infections and increased vaccination rates, new variants that pose significant public health concerns continue to emerge. Monitoring the efficacy of acquired immunity against these variants is crucial to guide our strategies for controlling the constantly evolving virus. Measurement of neutralizing activity elicited by vaccination or infection is being used to evaluate the efficacy of humoral immunity against emerging variants. Currently, the plaque reduction neutralization test (PRNT), which measures the reduction of cytopathic effects caused by authentic infectious SARS-CoV-2, is considered the “gold standard” assay for evaluating neutralization activity of antisera (2). However, this method is relatively labor intensive and not scalable, especially when testing the cross-neutralizing activity of vaccine- or infection-induced humoral responses against large panels of SARS-CoV-2 variants of concern (VOCs). To overcome this, we sought to develop a novel assay that could assess neutralizing activity by quantifying virus-induced cytopathic effects on target cells using chemiluminescence as an alternative to the plaque reduction method used in PRNT. This approach not only reduces the labor intensity of the assay but also enables scalability while preserving the other key features of PRNT.

**FIG 1:**
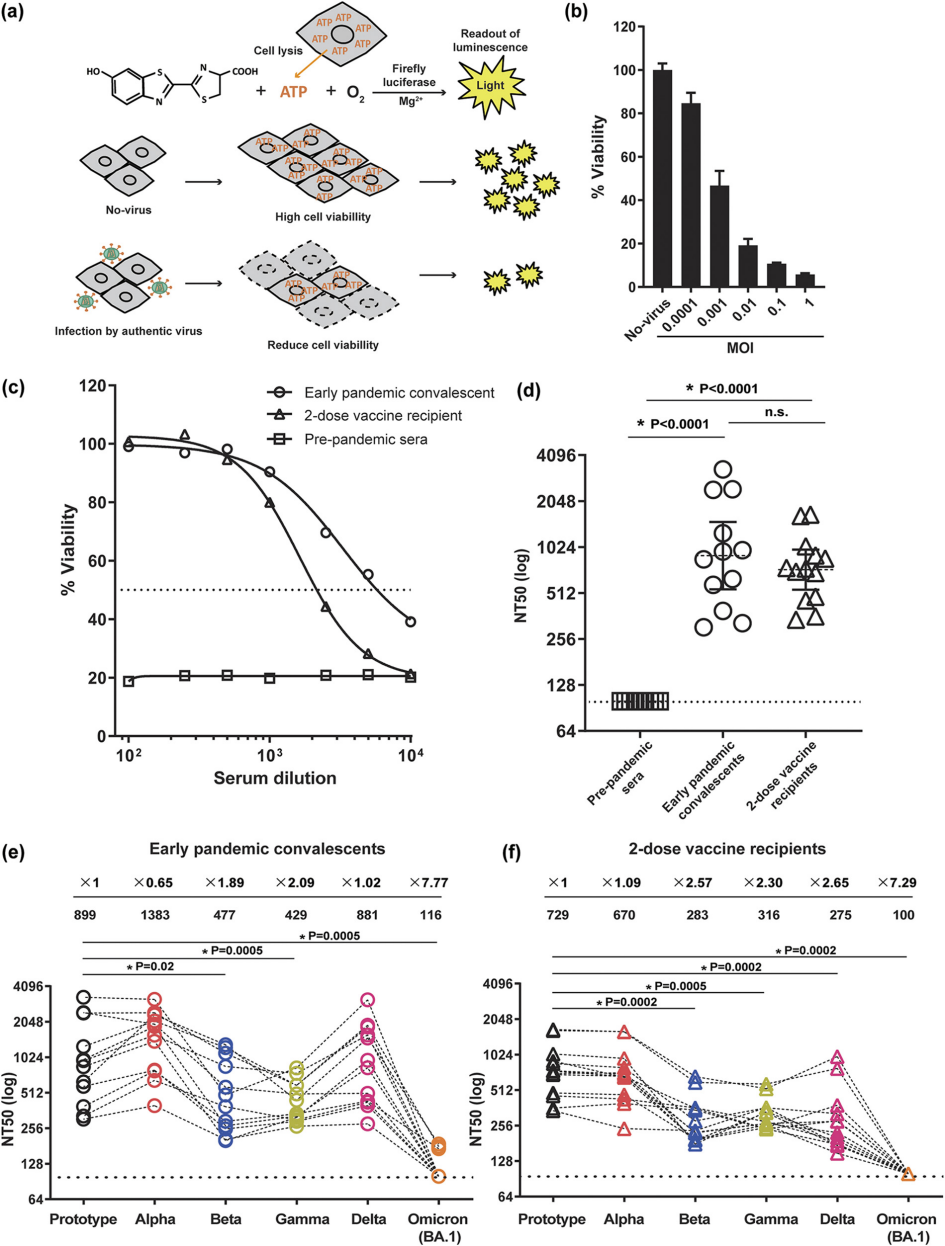
Evaluation of neutralizing capacity of antisera against clinically isolated coronaviruses. (a) Illustration of the principle of the SARS-CoV-2 cytopathic effect (CPE)-based luciferase assay. The live cell is the source of ATP in the luciferase reaction, and thereby luminescence intensity is proportional to the number of viable cells. When CPE occurs due to viral infection, the total amount of ATP is reduced. (b) The inoculum of the SARS-CoV-2 prototype was diluted at the indicated MOI in a serum-free buffer containing 20 mM HEPES, 1× minimal essential medium (MEM), and 1× nonessential amino acids (NEAA) and then exposed to Vero E6/TMPRSS2 cells (1 × 10^4^ cells) at 37°C that had been seeded on a 96-well plate. Forty-eight hours later, a chemiluminescence substrate (CellTiter-Glo 2.0 assay; Promega) containing 0.2% Triton X-100 was added and the luminescence intensity was determined by a plate reader (CentroXS3; Berthhold Technologies). The percentage of viability was set as 100% in the absence of virus infection. (c) Antiserum samples isolated from the indicated groups were prepared by 2-fold serial dilution starting from 100-fold dilution through 6,400-fold dilution in a serum-free dilution buffer. Equal volumes (25 μL) of the resultant antisera and the prototype virus inoculum (MOI = 0.01) were mixed at 37°C for 1 h. The resulting virus mixture was exposed to Vero E6/TMPRSS2 cells as described above. As a representative data set, the percentages of viability of three individuals are shown. The NT_50_ was interpolated using a 4-parameter nonlinear regression in GraphPad Prism 9.3.1. (d) The dot plot depicted here shows comparison of NT_50_s of prepandemic sera, early pandemic convalescents, and 2-dose vaccine recipients. The horizontal dashed line indicates the detection limit; the lowest serum dilution tested was 1:100. (e and f) Neutralization assays were performed with authentic SARS-CoV-2 of the prototype (Wuhan strain; MOI = 0.01), Alpha (MOI = 0.01), Beta (MOI = 0.01), Gamma (MOI = 1), Delta (MOI = 1), and Omicron (BA.1) (MOI = 0.1) viruses in early pandemic convalescents and 2-dose vaccine recipients. Each dot represents the NT_50_ value of an antiserum sample, determined as the mean of quadruplicate assays. The geometric mean and the fold reduction of neutralization activity relative to Wuhan strain are shown at the top. All NT_50_ values are listed in Table S2. The horizontal dashed line indicates the detection limit; the lowest serum dilution tested was 1:100. Statistical analysis was performed using the Wilcoxon matched-pair signed-rank test.

Based on the principle that the number of viable cells is proportional to the amount of ATP in the cell lysate (Fig. 1a), we postulated that determining cell viability in culture by quantifying ATP (CellTiter-Glo 2.0 assay; Promega) can be used as a measure of the SARS-CoV-2-induced cytopathic effect. To test this, we exposed increasing titers of SARS-CoV-2 (Wuhan strain; DDBJ accession ID no. LC528232) (see Table S1 in the supplemental material) to the target cells (Vero E6/TMPRSS2 cells). We observed a steady decline in cell viability with increasing virus titers corresponding to the cytopathic effects (CPEs) induced by the virus infection (Fig. 1b). Using this approach to quantify SARS-CoV-2-induced cytopathic effect, we aimed to develop an assay to quantitate neutralizing potency of sera from vaccinated and infected individuals. To validate the assay, we obtained three sets of serum samples: (i) 12 early pandemic convalescents (August to November 2020) from Kyushu Medical Center and Kyushu University Hospital, both in Fukuoka, Japan, and from Kumamoto University Hospital, Kumamoto, Japan; (ii) 13 healthy volunteers who received 2 doses of BNT162b2 (Pfizer-BioNTech) (April to June 2021); and (iii) 9 prepandemic samples from healthy volunteers obtained between 2016 and early 2019 (see Table S2 for participant information). The SARS-CoV-2 (Wuhan strain; multiplicity of infection [MOI] = 0.01) was preincubated with seven 2-fold serially diluted serum samples starting from a 100-fold dilution. Next, we exposed them to Vero E6/TMPRSS2 cells and allowed for a 48-h incubation period. While no protection was observed in prepandemic sera, the protection from virus-mediated cytopathic effect declined with the increased dilution of serum samples from both the early pandemic convalescents and the 2-dose vaccine recipients (Fig. 1c). The 50% neutralization titer (NT_50_) value was defined as the minimum serum dilution that gave >50% of cell viability. All sera with nonquantifiable NT_50_ values were assigned to the lowest plasma dilution factor (i.e., NT_50_ = 100) for plotting purposes and statistical analysis. The geometric mean NT_50_s of early pandemic convalescents and 2-dose vaccine recipients against the Wuhan strain were comparable but significantly higher than those of prepandemic samples (Fig. 1d). These results demonstrated that our chemiluminescence-based, cytopathic effect assay can specifically detect neutralizing activity associated with SARS-CoV-2 infection or vaccination.

Employing the assay, we determined the cross-reactive neutralizing capacity of antiserum samples from early pandemic convalescents and the 2-dose vaccine recipients toward a panel of SARS-CoV-2 VOCs, including Alpha (GISAID accession ID EPI_ISL_768526), Beta (EPI_ISL_1123289), Gamma (EPI_ISL_833366), Delta (EPI_ISL_2080609), and Omicron BA.1 (EPI_ISL_8559478) (Table S1). For each variant, the MOI that gave 15 to 20% viability was used for the evaluation of neutralizing sensitivity. Confirming the previously reported findings (3–6), the neutralizing capacity in both sets of samples was significantly reduced against Beta, Gamma, and Omicron/BA.1 (all at *P* ≤ 0.02) but maintained against Alpha, compared with the Wuhan strain (Fig. 1e). Interestingly, the neutralizing capacity against Delta was maintained in samples from the early pandemic convalescents but reduced in those from 2-dose vaccine recipients (Fig. 1e).

**FIG 2:**
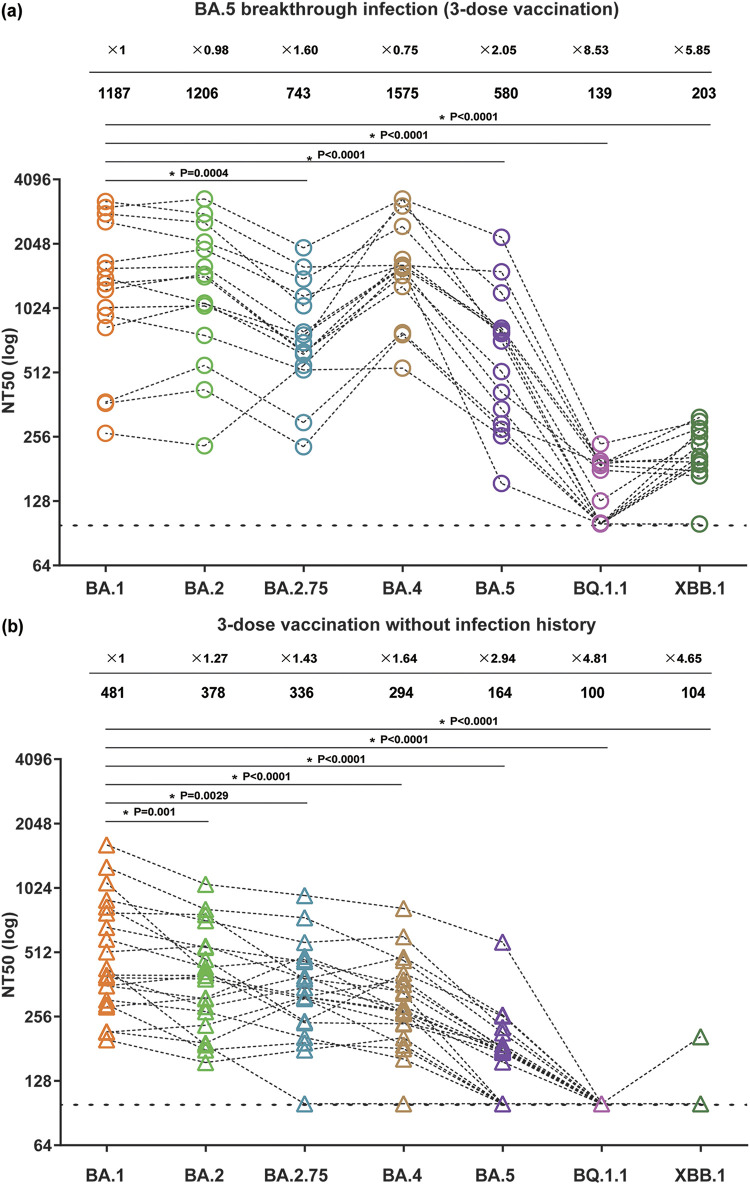
Neutralization against Omicron subvariants. Neutralization assays were performed in BA.5 breakthrough infection sera (a) and 3-dose vaccination sera without infection history (b) against Omicron subvariants BA.2 (MOI = 0.1), BA.2.75 (MOI = 0.1), BA.4 (MOI = 0.1), BA.5 (MOI = 1), BQ.1.1 (MOI = 0.1), and XBB.1 (MOI = 1). Assays with each serum sample were performed in quadruplicate to determine the NT_50_. Each dot represents one NT_50_ value, and the geometric mean and fold reduction of neutralization activity relative to BA.1 are shown at the top. All NT_50_ values are listed in Table S2. The horizontal dashed line indicates the detection limit; the lowest serum dilution tested was 1:100. Statistically significant difference between BA.1 and Omicron subvariants was determined by Wilcoxon matched-pair signed-rank test.

As of May 2022, the SARS-CoV-2 Omicron variants BA.4 and BA.5 had become widespread (7, 8), following the emergence of BA.1 in late 2021 and BA.2 in early 2022 (9, 10). Thereafter, descendants of BA.2 such as BA.2.75 and XBB.1, as well as a descendant of BA.5 named BQ.1.1, emerged and are now becoming predominant in certain countries (7). These Omicron variants have acquired far more mutations in Spike (S) than previous variants (8–10) and show substantial resistance against antisera obtained from individuals receiving 2 doses of mRNA vaccine (5, 6). Against the emerging Omicron variants, including XBB.1 and BQ.1.1, we sought to analyze the cross-reactive capacity of antisera isolated from individuals who received 3 doses of mRNA vaccines, as well as those who had BA.5 breakthrough infection after receiving 3-dose vaccination, using our chemiluminescence-based cytopathic effect assay. Fifteen individuals with BA.5 breakthrough infections from Kyushu Medical Center, Fukuoka, Japan, and Kumamoto University Hospital, Kumamoto, Japan, were recruited after obtaining written informed consent. In addition, we recruited 21 healthy volunteers who had received the 3-dose vaccination with BNT162b2 (Pfizer-BioNTech) or mRNA-1273 (Moderna) (February to March 2022) (see Table S2 for participant information). Then, antiserum samples were tested for cross-reactive neutralizing capacity against BA.1, BA.2 (EPI_ISL_12812500), BA.2.75 (EPI_ISL_13969765), BA.4 (EPI_ISL_13278440), BA.5 (EPI_ISL_12812500), BQ.1.1 (EPI_ISL_15579783), and XBB.1 (EPI_ISL_15669344) variants (Table S1). The geometric mean NT_50_s of the BA.5 breakthrough infection group against BA.1, BA.2, BA.2.75, BA.4, BA.5, BQ.1.1, and XBB.1 were 1,187, 1,206, 743, 1,575, 580, 139, and 203, respectively (Fig. 2a). Compared to BA.1, those against BA.2.75, BA.5, BQ.1.1, and XBB.1 were reduced by 1.6-fold, 2-fold, 8.5-fold, and 5.9-fold, respectively (all at *P* ≤ 0.004), but those against BA.2 and BA.4 were maintained (Fig. 2a). On the other hand, the geometric mean NT_50_s of the 3-dose vaccination group against BA.1, BA.2, BA.2.75, BA.4, BA.5, BQ.1.1, and XBB.1 were 481, 378, 336, 294, 164, 100, and 104, respectively (Fig. 2b). Those against BA.2, BA.2.75, BA.4, BA.5, BQ.1.1, and XBB.1 were reduced by 1.3-fold, 1.4-fold, 1.6-fold, 2.9-fold, 4.8-fold, and 4.6-fold, respectively, compared to BA.1 (all at *P* ≤ 0.001) (Fig. 2b). These results suggested that (i) BA.5 breakthrough infection after 3-dose vaccination increased the magnitude and breadth of cross-neutralizing capacity compared with vaccination only, (ii) among the Omicron subvariants, BQ.1.1 and XBB.1 exhibited the highest level of immune resistance, and (iii) the antiserum samples with strong neutralizing capacity against BA.1 also showed relatively strong cross-reactive neutralizing capacity against other Omicron subvariants. Collectively, our neutralization results support the idea that the newly emerging Omicron subvariants are continuing to increase their capability for immune resistance against vaccine- and/or infection-elicited humoral immunity. Of the Omicron subvariants tested, BQ.1.1 and XBB.1 exhibited the greatest resistance to both vaccine- and infection-elicited neutralization, suggesting the potential of these emerging variants to dethrone BA.5 as the dominant lineage in circulation.

In summary, we established a new scalable assay system using replication-competent, authentic SARS-CoV-2 isolated from nasal swabs for quantifying neutralizing activity of antisera. As an alternative to the plaque assay, it can be used for evaluation of neutralizing activity against constantly emerging variants.
