# Supplementary material for: Evaluation of Neutralizing Activity against Omicron Subvariants in BA.5 Breakthrough Infection and Three-Dose Vaccination Using a Novel Chemiluminescence-Based, Virus-Mediated Cytopathic Assay
Source: Microbiol Spectr. 2023 Jun 13;11(4):e00660-23. doi: 10.1128/spectrum.00660-23 (PMC10433814; doi:10.1128/spectrum.00660-23)
Supplement: Supplemental file 1 — Table S1. Download spectrum.00660-23-s0001.pdf, PDF file, 0.01 MB [file spectrum.00660-23-s0001.pdf]

Table S1. Clinically isolated SARS-CoV-2 strains

Pango Lineage	Virus name	DDBJ/GISAID Accession ID	Resource
Wuhan strain	SARS-CoV-2/Hu/DP/Kng/19-020	LC528232	Kanagawa Prefectural Institute of Public Health
B.1.1.7 (Alpha)	hCoV-19/Japan/QK002/2020	EPI_ISL_768526	National Institute of Infectious Diseases, Tokyo, Japan
B.1.351 (Beta)	hCoV-19/Japan/TY8-612-P1/2021	EPI_ISL_1123289	National Institute of Infectious Diseases, Tokyo, Japan
P.1 (Gamma)	hCoV-19/Japan/TY7-501/2021	EPI_ISL_833366	National Institute of Infectious Diseases, Tokyo, Japan
B.1.617.2 (Delta)	hCoV-19/Japan/TKYK01734/2021	EPI_ISL_2080609	Tokyo Metropolitan Institute of Public Health, Tokyo, Japan
B.1.1.529 (Omicron/BA.1)	hCoV-19/Japan/TKYX00012/2021	EPI_ISL_8559478	Tokyo Metropolitan Institute of Public Health, Tokyo, Japan
B.1.1.529 (Omicron/BA.2)	hCoV-19/Japan/TY40-385-P1/2022	EPI_ISL_9595859	National Institute of Infectious Diseases, Tokyo, Japan
B.1.1.529 (Omicron/BA.2.75)	hCoV-19/Japan/TY41-716/2022	EPI_ISL_13969765	National Institute of Infectious Diseases, Tokyo, Japan
B.1.1.529 (Omicron/BA.4)	hCoV-19/Japan/TY41-703/2022	EPI_ISL_13278440	National Institute of Infectious Diseases, Tokyo, Japan
B.1.1.529 (Omicron/BA.5)	hCoV-19/Japan/TKYS14631/2022	EPI_ISL_12812500	Tokyo Metropolitan Institute of Public Health, Tokyo, Japan
B.1.1.529 (Omicron/BQ.1.1)	hCoV-19/Japan/TY41-796/2022	EPI_ISL_15579783	National Institute of Infectious Diseases, Tokyo, Japan
B.1.1.529 (Omicron/XBB.1)	hCoV-19/Japan/TY41-795/2022	EPI_ISL_15669344	National Institute of Infectious Diseases, Tokyo, Japan

All virus strains were propagated with VeroE6/TMPRSS2 cells (JCRB1819) in 10% FBS/DMEM (Wako, cat# 041-29775) containing 1 mg/mL G418 (Nacalai Tesque), and plaque-forming unit (PFU) was determined
