# Supplementary material for: Evaluation of Neutralizing Activity against Omicron Subvariants in BA.5 Breakthrough Infection and Three-Dose Vaccination Using a Novel Chemiluminescence-Based, Virus-Mediated Cytopathic Assay
Source: Microbiol Spectr. 2023 Jun 13;11(4):e00660-23. doi: 10.1128/spectrum.00660-23 (PMC10433814; doi:10.1128/spectrum.00660-23)
Supplement: Supplemental file 2 — Table S2. Download spectrum.00660-23-s0002.pdf, PDF file, 0.1 MB [file spectrum.00660-23-s0002.pdf]

Table S2. Characteristics of participants at baseline.

Cohort	Donnor	Sex	Age (year)	History of COVID-19	Blood collection	days after onset	No. of doses	Dose			Time since last shot (days)	NT50											
								1	2	3		Wuhan strain	Alpha	Beta	Gamma	Delta	BA.1	BA.2	BA.2.75	BA.4	BA.5	BQ.1.1	XBB.1
Pre-pandemic donors	1	M	40	no	2016/12/7	N/A	N/A	N/A	N/A	N/A	N/A	100	N/A	N/A	N/A	N/A	N/A	N/A	N/A	N/A	N/A	N/A	N/A
	2	M	37	no	2016/12/14	N/A	N/A	N/A	N/A	N/A	N/A	100	N/A	N/A	N/A	N/A	N/A	N/A	N/A	N/A	N/A	N/A	N/A
	3	F	32	no	2017/1/29	N/A	N/A	N/A	N/A	N/A	N/A	100	N/A	N/A	N/A	N/A	N/A	N/A	N/A	N/A	N/A	N/A	N/A
	4	M	35	no	2019/5/17	N/A	N/A	N/A	N/A	N/A	N/A	100	N/A	N/A	N/A	N/A	N/A	N/A	N/A	N/A	N/A	N/A	N/A
	5	M	34	no	2019/5/21	N/A	N/A	N/A	N/A	N/A	N/A	100	N/A	N/A	N/A	N/A	N/A	N/A	N/A	N/A	N/A	N/A	N/A
	6	M	40	no	2019/5/22	N/A	N/A	N/A	N/A	N/A	N/A	100	N/A	N/A	N/A	N/A	N/A	N/A	N/A	N/A	N/A	N/A	N/A
	7	M	36	no	2019/5/27	N/A	N/A	N/A	N/A	N/A	N/A	100	N/A	N/A	N/A	N/A	N/A	N/A	N/A	N/A	N/A	N/A	N/A
	8	M	28	no	2019/5/27	N/A	N/A	N/A	N/A	N/A	N/A	100	N/A	N/A	N/A	N/A	N/A	N/A	N/A	N/A	N/A	N/A	N/A
	9	M	57	no	2019/5/28	N/A	N/A	N/A	N/A	N/A	N/A	100	N/A	N/A	N/A	N/A	N/A	N/A	N/A	N/A	N/A	N/A	N/A
Early pandemic convalescents	10	M	72	2020/7/23	2020/8/7	15	N/A	N/A	N/A	N/A	N/A	632.3	2339.6	1305	442.5	1489.7	188.1	N/A	N/A	N/A	N/A	N/A	N/A
	11	M	60	2020/7/28	2020/8/14	17	N/A	N/A	N/A	N/A	N/A	578.9	795.1	252.4	293.1	397.5	100	N/A	N/A	N/A	N/A	N/A	N/A
	12	M	60	2020/7/28	2020/8/19	22	N/A	N/A	N/A	N/A	N/A	391.3	783.8	292.9	339	434.5	171.7	N/A	N/A	N/A	N/A	N/A	N/A
	13	M	49	2020/8/4	2020/8/24	20	N/A	N/A	N/A	N/A	N/A	305.5	398.3	205.5	266.4	278.6	100	N/A	N/A	N/A	N/A	N/A	N/A
	14	M	78	2020/8/7	2020/8/27	20	N/A	N/A	N/A	N/A	N/A	976.8	1870.4	268.4	324.2	833.6	100	N/A	N/A	N/A	N/A	N/A	N/A
	15	F	49	2020/8/18	2020/8/31	13	N/A	N/A	N/A	N/A	N/A	1260	2076.8	1236.4	590.8	1847.2	100	N/A	N/A	N/A	N/A	N/A	N/A
	16	M	35	2020/8/15	2020/8/31	16	N/A	N/A	N/A	N/A	N/A	955.9	1576.3	854.8	748	1580	100	N/A	N/A	N/A	N/A	N/A	N/A
	17	M	74	2020/8/20	2020/9/2	13	N/A	N/A	N/A	N/A	N/A	324.8	651.1	389.8	300.7	425.8	100	N/A	N/A	N/A	N/A	N/A	N/A
	18	M	60	2020/8/28	2020/9/9	12	N/A	N/A	N/A	N/A	N/A	2428	2443	1107.8	590.4	1910.6	179.3	N/A	N/A	N/A	N/A	N/A	N/A
	19	M	81	2020/9/9	2020/9/23	14	N/A	N/A	N/A	N/A	N/A	851.1	1398.1	201.6	316.3	965.4	100	N/A	N/A	N/A	N/A	N/A	N/A
	20	M	71	2020/8/29	2020/9/25	27	N/A	N/A	N/A	N/A	N/A	2445	1952	569.9	499.4	506	100	N/A	N/A	N/A	N/A	N/A	N/A
	21	M	42	2020/11/14	2020/11/18	4	N/A	N/A	N/A	N/A	N/A	3299	3164.2	490.5	832	3142	100	N/A	N/A	N/A	N/A	N/A	N/A
2-dose vaccine recipients	26	F	60	no	2021/4/19	no	x2	BNT162b2	BNT162b2	N/A	10	901.9	656.7	192.6	329.6	173.9	100	N/A	N/A	N/A	N/A	N/A	N/A
	22	M	48	no	2021/4/20	no	x2	BNT162b2	BNT162b2	N/A	14	342.1	241.5	232	364.1	178.2	100	N/A	N/A	N/A	N/A	N/A	N/A
	23	M	34	no	2021/4/20	no	x2	BNT162b2	BNT162b2	N/A	14	747.3	698.2	192.4	246.2	381.2	100	N/A	N/A	N/A	N/A	N/A	N/A
	24	M	55	no	2021/4/23	no	x2	BNT162b2	BNT162b2	N/A	14	359.7	394.5	346.6	239.4	177.5	100	N/A	N/A	N/A	N/A	N/A	N/A
	25	M	55	no	2021/4/23	no	x2	BNT162b2	BNT162b2	N/A	14	860.9	792.3	280.7	362.5	323.4	100	N/A	N/A	N/A	N/A	N/A	N/A
	27	M	35	no	2021/5/6	no	x2	BNT162b2	BNT162b2	N/A	14	1636	1604.9	666.7	526	984.3	100	N/A	N/A	N/A	N/A	N/A	N/A
	28	F	38	no	2021/5/10	no	x2	BNT162b2	BNT162b2	N/A	13	741.5	668.1	359.3	240.5	280.4	100	N/A	N/A	N/A	N/A	N/A	N/A
	29	M	33	no	2021/5/10	no	x2	BNT162b2	BNT162b2	N/A	13	458.6	429.8	361.8	275.9	190.6	100	N/A	N/A	N/A	N/A	N/A	N/A
	30	F	57	no	2021/5/10	no	x2	BNT162b2	BNT162b2	N/A	10	1671	1601	595.1	575.4	782.3	100	N/A	N/A	N/A	N/A	N/A	N/A
	31	F	18	no	2021/6/23	no	x2	BNT162b2	BNT162b2	N/A	21	707.7	717.5	221.5	266.5	223.6	100	N/A	N/A	N/A	N/A	N/A	N/A
	32	F	24	no	2021/6/29	no	x2	BNT162b2	BNT162b2	N/A	22	693.3	671.3	224.6	266.7	277.9	100	N/A	N/A	N/A	N/A	N/A	N/A
	34	M	23	no	2021/6/29	no	x2	BNT162b2	BNT162b2	N/A	22	1036	949.8	178.6	334.1	202.9	100	N/A	N/A	N/A	N/A	N/A	N/A
	33	F	25	no	2021/6/30	no	x2	BNT162b2	BNT162b2	N/A	23	483.7	466.8	195.9	260.1	149.1	100	N/A	N/A	N/A	N/A	N/A	N/A
BA.5 breakthrough infection (3-dose vaccination)	35	M	40	2022/7/19	2022/8/3	15	x3	BNT162b2	BNT162b2	mRNA-1273	175	N/A	N/A	N/A	N/A	N/A	1334.2	1432.7	634.6	1453.4	516.6	100	271.5
	36	F	56	2022/7/20	2022/8/3	14	x3	BNT162b2	BNT162b2	BNT162b2	103	N/A	N/A	N/A	N/A	N/A	366.9	425.2	230	768.5	277.5	100	197.1
	37	M	26	2022/7/13	2022/8/3	21	x3	mRNA-1273	mRNA-1273	BNT162b2	130	N/A	N/A	N/A	N/A	N/A	945.8	762.9	526.4	537.1	258.5	100	234.8
	38	M	45	2022/7/16	2022/8/10	25	x3	BNT162b2	BNT162b2	BNT162b2	239	N/A	N/A	N/A	N/A	N/A	2594.2	2088.1	1398.9	2467.4	789.2	128.3	255.5
	39	M	38	2022/7/19	2022/8/12	24	x3	BNT162b2	BNT162b2	BNT162b2	149	N/A	N/A	N/A	N/A	N/A	372.1	552.5	298.5	786.1	294.5	192.2	205.7
	40	M	30	2022/6/25	2022/8/12	48	x3	mRNA-1273	mRNA-1273	mRNA-1273	152	N/A	N/A	N/A	N/A	N/A	1247.8	1474.4	640.2	1292.4	345.6	100	100
	41	F	46	2022/7/30	2022/8/12	13	x3	mRNA-1273	mRNA-1273	mRNA-1273	241	N/A	N/A	N/A	N/A	N/A	1417	1075.4	765.9	1571.5	413.4	178.7	167.8
	42	M	63	2022/7/19	2022/8/20	32	x3	mRNA-1273	mRNA-1273	mRNA-1273	157	N/A	N/A	N/A	N/A	N/A	3229.8	2818.7	1591.6	1624.1	825	197.4	195.1
	43	F	51	2022/7/28	2022/8/20	23	x3	mRNA-1273	mRNA-1273	mRNA-1273	162	N/A	N/A	N/A	N/A	N/A	3025.7	3320	1961.9	3316.6	2196.7	236.7	301.8
	44	M	43	2022/8/12	2022/8/20	8	x3	mRNA-1273	mRNA-1273	mRNA-1273	154	N/A	N/A	N/A	N/A	N/A	265.9	232.1	552.5	1571.5	154.8	100	190
	45	F	46	2022/7/23	2022/8/23	31	x3	mRNA-1273	mRNA-1273	mRNA-1273	231	N/A	N/A	N/A	N/A	N/A	1671.7	1926.3	1164.1	1624.1	1513.7	190.3	316
	46	M	64	2022/8/8	2022/8/24	16	x3	BNT162b2	BNT162b2	BNT162b2	169	N/A	N/A	N/A	N/A	N/A	1030.3	1047.8	706	3316.6	717.7	101	207
	47	F	62	2022/8/9	2022/8/24	15	x3	BNT162b2	BNT162b2	BNT162b2	153	N/A	N/A	N/A	N/A	N/A	830.5	1080.4	622.7	1729.1	778.6	100	100
	48	F	33	2022/8/2	2022/8/24	22	x3	BNT162b2	BNT162b2	BNT162b2	152	N/A	N/A	N/A	N/A	N/A	1567.6	1599.3	792.5	1585.5	803.1	188.1	177.4
	49	M	33	2022/8/1	2022/8/27	26	x3	BNT162b2	BNT162b2	mRNA-1273	137	N/A	N/A	N/A	N/A	N/A	2828.6	2575	1049.8	3075.8	1205.9	189.7	280.2
3-dose vaccine recipients	50	M	56	no	2022/2/19	no	x3	BNT162b2	BNT162b2	mRNA-1273	24	N/A	N/A	N/A	N/A	N/A	897.2	720.1	568.9	605.9	227.6	100	100
	51	M	39	no	2022/2/19	no	x3	BNT162b2	BNT162b2	mRNA-1273	24	N/A	N/A	N/A	N/A	N/A	199.4	156	178.5	203.8	100	100	100
	52	F	67	no	2022/2/22	no	x3	BNT162b2	BNT162b2	BNT162b2	15	N/A	N/A	N/A	N/A	N/A	834.2	474.6	243.5	407.9	257.7	100	100
	53	M	39	no	2022/3/3	no	x3	BNT162b2	BNT162b2	mRNA-1273	24	N/A	N/A	N/A	N/A	N/A	781.7	768.4	383.1	486	260.8	100	206.1
	54	F	23	no	2022/3/7	no	x3	BNT162b2	BNT162b2	mRNA-1273	40	N/A	N/A	N/A	N/A	N/A	216.3	233.3	315.9	269.6	100	100	100
	55	F	29	no	2022/3/7	no	x3	BNT162b2	BNT162b2	mRNA-1273	40	N/A	N/A	N/A	N/A	N/A	1081.3	416	239.6	236.8	174	100	100
	56	F	23	no	2022/3/7	no	x3	BNT162b2	BNT162b2	mRNA-1273	40	N/A	N/A	N/A	N/A	N/A	356.2	310.1	395.7	264.3	156.9	100	100
	57	F	25	no	2022/3/7	no	x3	BNT162b2	BNT162b2	mRNA-1273	40	N/A	N/A	N/A	N/A	N/A	356.4	398.2	317.1	380.9	180.7	100	100
	58	F	24	no	2022/3/8	no	x3	BNT162b2	BNT162b2	mRNA-1273	41	N/A	N/A	N/A	N/A	N/A	389.4	383.9	310.8	239	189.6	100	100
	59	M	24	no	2022/3/8	no	x3	BNT162b2	BNT162b2	mRNA-1273	41	N/A	N/A	N/A	N/A	N/A	397.6	285	349.6	286.1	176.9	100	100
	60	M	24	no	2022/3/8	no	x3	BNT162b2	BNT162b2	mRNA-1273	41	N/A	N/A	N/A	N/A	N/A	217.6	192.5	310.6	188.4	100	100	100
	61	M	23	no	2022/3/8	no	x3	BNT162b2	BNT162b2	mRNA-1273	41	N/A	N/A	N/A	N/A	N/A	283.9	313.5	490.8	329	100	100	100
	62	F	24	no	2022/3/9	no	x3	BNT162b2	BNT162b2	mRNA-1273	42	N/A	N/A	N/A	N/A	N/A	1629	1064.7	942.9	820.1	570.8	100	100
	63	M	22	no	2022/3/9	no	x3	BNT162b2	BNT162b2	mRNA-1273	42	N/A	N/A	N/A	N/A	N/A	306.1	269.8	204.5	162.1	100	100	100
	64	M	22	no	2022/3/9	no	x3	BNT162b2	BNT162b2	mRNA-1273	42	N/A	N/A	N/A	N/A	N/A	669.2	540.3	463.5	361.5	183.7	100	100
	65	M	24	no	2022/3/10	no	x3	BNT162b2	BNT162b2	mRNA-1273	43	N/A	N/A	N/A	N/A	N/A	399.9	398.5	316.7	273.3	175	100	100
	66	F	23	no	2022/3/10	no	x3	BNT162b2	BNT162b2	mRNA-1273	43	N/A	N/A	N/A	N/A	N/A	514.4	547.2	385.6	336	177.4	100	100
	67	M	23	no	2022/3/11	no	x3	BNT162b2	BNT162b2	mRNA-1273	44	N/A	N/A	N/A	N/A	N/A	1279	812	743.3	469.1	214.8	100	100
	68	M	22	no	2022/3/11	no	x3	BNT162b2	BNT162b2	mRNA-1273	44	N/A	N/A	N/A	N/A	N/A	291.1	178.7	192.9	181.2	100	100	100
	69	M	22	no	2022/3/11	no	x3	BNT162b2	BNT162b2	mRNA-1273	44	N/A	N/A	N/A	N/A	N/A	429.3	188.8	100	100	100	100	100
	70	M	23	no	2022/3/11	no	x3	BNT162b2	BNT162b2	mRNA-1273	44	N/A	N/A	N/A	N/A	N/A	589.6	435.3	473.8	267.2	177	100	100
